# Roles of metacognition and achievement goals in mathematical modeling competency: A structural equation modeling analysis

**DOI:** 10.1371/journal.pone.0206211

**Published:** 2018-11-06

**Authors:** Riyan Hidayat, Hutkemri Zulnaidi, Sharifah Norul Akmar Syed Zamri

**Affiliations:** Department of Mathematics and Science Education, Faculty of Education, University of Malaya, Kuala Lumpur, Malaysia; Jiangsu Normal University, CHINA

## Abstract

This study investigates the relationship between metacognition and achievement goals which may influence mathematical modeling competency in students of mathematics education programs. The current study employs 538 students of mathematics education program; 483 (89.8%) of whom are male and 55 (10.2%) are aged from 18 years old to 22 years old. The study follows a correlational research design to investigate and measure the degree of relationship amongst mathematical modeling competencies, achievement goals and metacognition. Results indicate that achievement goals and metacognition positively influence mathematical modeling competency. Moreover, four metacognition dimensions including awareness, planning, cognitive strategy and self-checking are positive partial mediators because they increase the association between achievement goals and mathematical modeling competency. In conclusion, metacognition and achievement goals positively affect students’ mathematical modeling competency.

## Introduction

Educational researchers have highlighted several key benefits of modeling competencies to the study of intricacy and to modern science [1–3], particularly social science (e.g. economics, information systems, finance, business, education and the arts). Students in high education levels with these competencies are assumed to successfully conduct a research because these competencies involve a rigorous scientific procedure [4]. Surprisingly, mathematical analysis and modeling are widely used by policy makers and industrialists for guidance and decision making [5]. However, the application of a mathematical modeling process in mathematical classrooms is a new challenge for primary school [6], secondary school [7–9] and even higher education students [3, 10, 11], particularly mathematics education program students [12–14]. Previous studies have emphasised that students have difficulty interpreting real contexts into mathematical contexts [9, 15, 16]. For example, in a study conducted by Blomhøj and Kjeldsen [10], students encountered problems on mathematising the expression ‘proportional to the square of population size’ before finding formula.

Modeling behaviour is complex, persistent and repetitive. Few studies have been conducted on understanding factors that may influence students’ mathematical modeling competency [17–21]. A few potential influencing factors are goal oriented [22, 23] and metacognition [16, 24–26]. Zimmerman and Campillo [27] argued that students must not only have sufficient knowledge in solving a complex problem but also have strong motivation and personal resourcefulness to carry out the challenge. Goal oriented [28] and metacognition [29] are regarded as part of the definition of mathematical modeling competency [30]. Therefore, goal-oriented and metacognitive competencies are no longer regarded as positive side impacts on but are significant constituents of mathematical modeling competency. To our knowledge, the effects of achievement goals and metacognition on students’ mathematical modeling competency have not been examined.

In this study, we proposed to extend existing mathematical modeling competency literature by discussing these associations in the context of real-life problems. This study investigated the relationship between metacognition and achievement goals which may influence the mathematical modeling competency in students of mathematics education programs. This research also focused on the direct and indirect effects of the relationship between achievement goals and mathematical modeling competency. The indirect effects are the mediating effects of four metacognition dimensions, namely, awareness, planning, cognitive strategy and self-checking. The active agents of metacognition have received less attention in existing literature [31]. Thus, the examination of four metacognition sub-constructs is valuable. The research questions that guided the study are as follows:

1. Do metacognition and achievement goals directly affect mathematical modeling competency?
2. Do the four metacognition sub-constructs play a mediating role in achievement goals and mathematical modeling competency?

## Literature review

### Mathematical modeling competency

Modeling, also known as mathematising or mathematisation [32, 33], refers to a process of organising representational descriptions [34] within which symbolic means and model formal structures or formal structures emerge [35, 36]. This process is usually considered a group activity [37]. However, a different view exists when mathematical modeling is implemented in curricular debate. A mathematical modeling perspective states that it integrates data into examples [38], whereas others believe that mathematical modeling is a conclusion in itself and is not a means to reach other mathematical learning ending.

Mathematical modeling competency is defined in three different parts of competence: cognitive, affective and metacognitive competencies [30]. Affective and metacognitive competencies are no longer considered as merely positive side impacts of mathematical modeling but became significant constituent parts of mathematical modeling. In cognitive competencies, modeling competency is closely associated with the definition of the modeling process [29, 39]. For example, Blum [40] regarded mathematical modeling competency as the capability of identifying pertinent questions, variables, relations or assumptions in a given real-world situation by mathematising these into mathematics and interpreting and validating the solution of the resulting mathematical problem in relation to the given situation. They also conceptualised mathematical modeling competency as the capability of analysing or comparing given models by exploring the assumptions being made and checking the properties and scope of a given model. Metacognition associates with the factors that support cognition, whereas affective competencies are related to beliefs in mathematics, the nature of problems and the value of mathematics in solving real problems [30]. The latter definition is line with that of Maaß [29], who included goal oriented or affective goal [28] and the willingness to situate these into action.

The mathematical modeling competency in the current research was evaluated using eight elements: simplifying assumptions; clarifying the goal; formulating the problem, assigning variables, parameters and constants; formulating mathematical statements; selecting a model; interpreting graphical representations; and relating back to the real situation [41]. This procedure is referred to as micro assessment [37]. In this research, the effects of mastery goal orientation and metacognition on mathematical modeling competency were examined.

### Relationship between metacognition and mathematical modeling competencies

Metacognition, which involves psychological and cognitive concepts [42], refers to people’s knowledge or cognitive activity on their own cognitive processes and products or anything associated with them [43, 44]. O’Neil and Abedi, [45] operationally interpret students’ metacognitive inventory as a construct involving the following sub-scales or sub-behaviors: planning, self-checking, cognitive strategy, and awareness. This construct of metacognition is in line with the definition of metacogcinition in prior literature [46–48]. The term of planning is that students have to have a goal (either assigned or self-directed) and a plan to reach the goal; self-checking refers to where students necessitate a self-checking mechanism to observe goal achievement; awareness is that the process is conscious to the individual. Interestingly, the term of cognitive strategy used in the current research rather than strategy only is that cognitive strategy refer to reflection on cognitive abilities and activities during the accomplishment of an assignment (for example, determining on the several ways of solving mathematical modeling task).

Metacognition is also classified as higher order thinking [49] that entangles active control over the cognitive processes engaged in learning process [50]. Metacognition is also an important strategy connected to mathematics achievement [51–55] and problem-solving skills [56]. Many studies have confirmed the importance of metacognition in improving mathematical modeling competency. Metacognition influences the development of modeling strategies of students when the effects of four metacognitive dimensions, namely, awareness, planning, cognitive strategy and self-checking, are considered [21]. Students who conduct better self-check show higher growth in their modeling competencies than those who are less skillful in self-checking. Cognitive strategy and planning skills also mediate development in modeling competencies. After some experience with modeling is achieved, students with increased skills in the two metacognitive dimensions carried out modeling better. However, the cognitive and metacognitive activities did not happen sequentially in the process; they established simultaneous and intertwined process in modeling, through which planning activities are most common, whereas prediction activities are least common [57].

Modeling problems are known as difficult tasks. Thus, metacognitive strategies are valuable in a modeling-oriented classroom. Metacognition is activated in difficult tasks [58]. Pennequin, Sorel, Nanty and Fontaine [59] found that metacognition boosts the use of general strategies such as task analysis, problem representation, prediction, planning, monitoring, checking, reflection and evaluation of success. The use of metacognitive strategies in classrooms enables students to be sensitive in understanding a problem appropriately and making few errors in the learning process, thereby improving their self-regulation skills and self-confidence [60]. However, numerous studies have also indicated that metacognition provides a diverse total of variance towards mathematics performance. For example, metacognitive knowledge and skills explain 42% of the total variance of mathematics achievement [61], whereas metacognitive experience merely explains 4% of the total variance of mathematics achievement [54].

Metacognition, which comes from studies in other domains, positively impacts problem-solving skills [62]. Although students spent little time organising or representing information (drawing/summarising) and even less time reviewing the progress and results of their performance, distinctions are found between accurate and inaccurate students in the metacognitive process during solving math problems [62]. They also discovered that accurate students pay significant attention to time planning (especially drawing and summarising); thus, they do not assess their progress and results. Interestingly, metacognitive training is mainly useful to low achievers as it allows them to progress and solve the same number of tasks [63].

Students regularly assisted with metacognitive and self-reflective activities focused on learning deeply and engaged and motivated throughout the study [63]. However, the role of metacognition in the mathematics problem differs for students with and without learning difficulties. Metacognition does not work well with learning difficulties [64] even when correlated to the mathematics problem [65]. For example, students with learning difficulties indicate a much lower mean score in recognising the sequence of steps to solve the tasks than those without learning difficulties [65].

### Relationship between achievement goals and mathematical modeling competencies

Achievement goals involve the purposes [66] or cognitive-dynamic manifestations [67] of achieving, developing or demonstrating high rather than low ability [68]. Purposes used in the conceptualisation of achievement goals are reasons behind engaging in achievement behaviour (to develop or demonstrate competence) and the objective pursued whilst engaging in achievement behaviour (objective/intrapersonal or normative competence) [69]. Different frameworks of achievement goal theory are available [66, 68, 70]. However, the six-component model of goal orientation, which involves task approach, task-avoidance, self-approach, self-avoidance, other-approach and other-avoidance [69], is the latest framework used in the current study. Elliot et al. [69] included task- and self-based goals under a single concept in which both exert an evaluative standard, such as mastery goal. Other-based goals are a direct analogue of performance goal. Competence in mastery and performance is conceptualised as approach or avoidance. Mastery-oriented goals (i.e. mastery and performance goals) are related to positive educational results, whereas avoidance goals (i.e. mastery- and performance-avoidance goals) often influence negative outcomes [71].

The positive relationship between achievement goals and mathematical modeling competency is obtained from considerable research in other domains. Previous studies have demonstrated that achievement goals correlate positively with students’ academic achievement [72–74] and problem-solving success [75]. Thus, students with high achievement goals should also have high performance in mathematical modeling competency. However, we could not find a research that documented the relationship between students’ mathematical modeling competency and achievement goals.

Modeling is usually regarded as a group activity [37]. Thus, mastery goals are useful. Mastery goals influence student relations, such as teacher–student relations, peer inclusion, peer conflict [76] and interest in the activity [77]. Mastery-oriented students are discovered to assess collaboration with respect to its contribution to learning, friendship and class cohesion and to be ready to collaborate with peers regardless of their social group membership [78]. Hagstrom and White [79] reported that success in solving problems is significantly linked to shared talk. This deduction reflects the importance of socially shared talk in the development of problem-solving strategies. Ferri and Lesh [80] reported that the modeling cycle can be managed goal orientedly if students have learned to talk and reflect on mathematical concepts and their own means of understanding mathematics.

For the learning task achievement, students in mastery goal environment concentrate on self-referent standards and the manageability and positive value of the learning task [81]. Mastery goal and a high level of involvement with a creative task are useful for students in reflecting on problem-solving strategies [51]. Stillman, Brown and Galbraith [82] found that students’ motivation for choice of real-world problematic situation to model, decision making on an approach and reporting of findings are particularly mastery-oriented rather than performance-oriented. Belenky and Nokes-Malach [83] highlighted that the mastery goal construct also allows students to develop knowledge, which is sufficiently conceptual and abstract, and transform it to a new problem-solving situation. Similarly, mastery goal is positively related to deep learning [84–86] and effective strategy use [66, 74].

Students who have mastery goal orientation can understand problems in several manners, devise a plan, carry out the plan and recall [75]. This skill is important in executing mathematical modeling tasks. For example, an effective and successful approach to mathematical modeling is the appropriate consideration of some relevant and irrelevant information in task requirements. Students need to plan what they should execute in solving a problem. Given that the planning stage refers to reading task materials, activating prior knowledge and motivational beliefs and forming initial goals for problem representation [87], goal orientation can be activated. Lazonder and Rouet [88] demonstrated that students with mastery goal orientation may strongly develop implicit goals in various text sections for problem representation.

Mastery goal consists of task- and self-based goals. However, Elliot et al. [69] indicated that task- and self-based competencies are not always appropriate towards all situations and should be separated into two entities depending on certain circumstances. Task-based goals predict academic self-concept [89], exam performance [90], perceived competence [73] and material absorption in class [69]. Self-based goals are unrelated to material absorption [69] and perceived competence [73]. Self-based and self-avoidance goals require abundant help-seeking [91]. However, self-based goal also predicts academic self-concept [89].

Performance goals (maladaptive) are classified by challenge avoidance and minimal perseverance in face of difficulty [68, 92]. The application of a performance goal in mathematical lessons is assumed to generate susceptibility to a ‘helpless’ scheme of reactions in accomplishment settings [67], such as a choice for simple or complicated tasks, withdrawal of effort in face of failure and mitigation of task enjoyment. Thus, performance goals predict surface strategy [86]. Performance goals, which refer to other-based goals, can predict positive learning outcomes [71]. However, this concept presents an insignificant relationship with CGPA [93, 94]. Further evidence comes from negative correlations between other-approach goals and exam performance [90], and students with other-approach goals necesitate considerable help-seeking [91]. Empirical studies have also indicated that other-avoidance goals are negatively associated with GPA [95].

### Relationship between achievement goals and metacognition

A robust goal intention does not ensure the achievement goals because students may fail to deal effectively with self-regulatory problems during goal striving [96]. Hence, goal orientation, which is a phase and sub-process of self-regulation, aims to integrate metacognitive processes (e.g. planning and strategy use) and social cognitive motives [27]. Metacognition is an important strategy that is connected to learning results [97, 98]. Many researchers have also indicated that goal orientation is closely associated with students’ metacognition [94, 99–101].

Zafarmand [102] found that, amongst three sub-constructs of goal orientation, mastery goal positively affects metacognitive awareness planning and monitoring, whereas performance goal is negatively related to metacognition. Students with good mastery goals are more likely to hold better metacognition than those with performance goals. Similarly, students’ views of mastery and performance goals indicated significant relationships with metacognitive self-regulation [103]. However, when mediated by learning style and self-efficacy, mastery and performance goals exhibited insignificant or no relationship with metacognition [104]. Moreover, students with performance-avoidance goals became involved in low levels of metacognitive activity [105]. Hence, we hypothesised that achievement goals, which involve mastery and performance goals, would positively affect students’ metacognition.

Metacognition of students mediates mastery goals and academic success [93, 94]. Students’ characteristics related to the mastery goal orientation can be self-regulated using self-monitoring and organizational strategies and are adaptive to failing in particular tasks [106]. Bonnett et al. [51] found that mastery goal orientations enhance metacognitive reflection on learning strategies and increase engagement [99]. Mathematics engagement involves the simultaneous recruitment of motivational and affective structures to guide sustained, productive learning behavior. However, the mediating effects of metacognition on mastery and performance goals differ. Coutinho [107] confirmed the partial mediator role of metacognition in mastery goals and academic success but no mediating effect is found between performance goals and academic success. Metacognition should influence academic success according to prior research. Thus, we hypothesized that metacognition indirectly affects mathematical modeling competency. The indirect influences in the this research are the mediating effects of four metacognition dimensions, namely, awareness, planning, cognitive strategy and self-checking, on achievement goals and mathematical modeling competency.

## Summary of the hypotheses

The three main hypotheses of this study are as follows:

Hypothesis 1. There will be no significant relationships between achievement goals and mathematical modeling competency.
Hypothesis 2. There will be no significant relationships between four metacognition dimensions, namely, awareness, planning, cognitive strategy and self-checking and mathematical modeling competency
Hypothesis 3. The Relationships between achievement goal and mathematical modeling competency will not be mediated by awareness, planning, cognitive strategy and self-checking.

## Method

### Participants and procedure

The study was approved by *Dinas Penanaman Modal dan Pelayanan Terpadu Satu Pintu* (DPMPTSP or Department of Investment and Integrated One Stop Services, Riau Province Government—Indonesia). The first approval letter from the University of Malaya, Jalan Universiti, 50603 Kuala Lumpur, Wilayah Persekutuan Kuala Lumpur—Malaysia, was sent to DPMPTSP and the agency, in return, sent this approval letter together with thier own consent letter to three research sites in Indonesia—Universitas Riau, Universitas Islam Negeri Sultan Syarif Kasim and Universitas Islam Riau, introducing both the intent of the project and the purpose of the study. Finally, the researcher administered survey to participating university ranging from one to two month timescale [108].

The population of this research was composed of students of a mathematics education program in Indonesia. These students were selected because of their mathematics course and modeling experiences commonly found amongst mathematics education programs. For example, they registered for some advanced courses, such as calculus, geometry, linear algebra, linear program and statistics. As a result, they were assumed to implicitly learn the process of mathematical modeling competency. This study selected groups rather than individuals; thus, cluster random sampling was suitable [108]. The current study employed 538 students of a mathematics education program in Riau Province, Indonesia. The female participants were 483 (89.8%), whereas the male participants were 55 (10.2%) with ages ranging from 18 years old to 22 years old. The gender disproportion in the departments of mathematics education program resulted in a large proportion of female participants. The academic year of the targeted students were the first year until the fourth year in school year 2017–2018. However, the current research only used the first, second and third years, because the fourth academic year students were in practical session. The students enrolled in the first academic year were 133 (24.7%), those in the second academic year were 223 (41.4%) and those in the third academic year were 182 (33.8%). Participants were invited to take part in the research by providing them a letter covering information about the aim of the study, tasks involved, the advantage and risks involved in following this research, and the confidentiality of their answers. All students from selected universities completed the survey, which was voluntary, during lecture hours. They also completed the questionnaire covering 22 items in mathematical modeling test, 20 items in metacognitive inventory and 12 items in the 3 × 2 achievement goal questionnaire. All classes of the mathematics education program took 45 min to 60 min to answer all questions.

### Measures

The translation preciseness of the original questionnaire was confirmed using back translation. Before the items were piloted, the questionnaire was translated from English by the authors and then translated back into Indonesian language by a bilingual language expert. The Indonesian version of the questionnaire indicates a similar factor structure to the original questionnaire. The three types of questionnaire were a mathematical modeling test constructed by Haines et al. [41], the 3 × 2 achievement goals adopted from Elliot et al. [69] and metacognitive inventory by O’Neil and Abedi [45]. The following sections begin with a mathematical modeling test, the 3 × 2 achievement goal questionnaire and then proceeds with sections on metacognitive inventory. All these instruments were piloted before the actual study was conducted.

#### Mathematical modeling competency test

The first instrument employed in this study was a mathematical modeling test containing real-world situations to investigate students’ mathematical modeling competency. The mathematical modeling test was originally developed by Haines et al. [41]. Firstly, the mathematical modeling test only measured 6 mathematical modeling competencies using 12 multiple-choice questions (5 alternative choices). They involved simplifying assumptions; clarifying the goal; formulating the problem; assigning variables, parameters and constants; formulating mathematical statements; and selecting a model. Competencies in mathematical modeling were then extended containing graphical representations and exploring real and mathematical world connections [37, 109]. However, it did not include competencies in solving mathematics, refining a model and reporting [41]. Students who correctly answered each multiple-choice format in the instrument were given 2 points, those with 1 or several partial correct answers were given 1 point and those with one incorrect answer were given 0 point. A total of 22 questions were used in the mathematical modeling test (maximum score is 44).

The reliability of the questions for mathematical modeling test was determined by discriminant index, difficulty index and Cronbach’s alpha by utilising the ANATES4 software. The value of the discrimination index for each item of the mathematical modeling test ranges from 24.55% to 57.27%. Therefore, several items are fairly good (two items) discrimination index, good (13 items) and very good (seven items). The value of the difficulty index for each item of the mathematical modeling test is between 31.36% and 56.82%. Therefore, all items possess a difficulty index at a moderate level. The reliability value of the mathematical modeling test (0.82) is good [110]. Hence, in the research, each item of the mathematical modeling competency was retained to examine the mathematical modeling competency of students.

#### The 3 × 2 achievement goal questionnaire

The questionnaire used was adopted from Elliot et al. [69], and it consists of six sub-constructs classified into mastery goals including task-approach, task-avoidance, self-approach and self-avoidance goals, as well as performance goals such as other-approach and other-avoidance goals. Each sub-construct contains three items (task-approach goal, ‘to obtain several questions right on the exams in this class’; task-avoidance goal, ‘to avoid incorrect answers on the exams in this class’; self-approach goal, ‘to perform better on the exams in this class than I have done in the past on these types of exams’; self-avoidance goal, ‘to avoid doing worse on the exams in this class than I normally do on these types of exams’; other-approach goal, ‘to outperform other students on the exams in this class’; and other-avoidance goal, ‘to avoid doing worse than other students on the exams in this class’). The questionnaire comprises of 18 questions measured on seven-point Likert-type scale, which reflect four sub-constructs. A 7-point scale ranging from 1 (‘strongly disagree’) to 7 (‘strongly agree’) was used in the 3 × 2 achievement goal questionnaire [111].

In the current research, reliability values of some scales exceeded the desirable standard of 0.70 (task-approach goal, *α* = 0.90; task-avoidance goal, *α* = 0.93; self-approach goal, *α* = 0.94; self-avoidance goal, *α* = 0.97; other-approach goal, *α* = 0.90; and other-avoidance goal, *α* = 0.88). All the CR values for the sub-construct achievement goals ranged from 0.88 to 0.97, which exceeded the desirable standard of 0.6. Therefore, a high internal consistency was obtained. Moreover, the average variance extracted (AVE) for the six latent variables ranged from 0.71 to 0.92, which also exceeded the common cut-off value of 0.5. Therefore, this study exhibited acceptable discriminant validity.

#### Metacognitive inventory questionnaire

The third instrument employed in this study was the metacognitive inventory to investigate the metacognition of students. The metacognitive inventory was originally constructed by O’Neil and Abedi [45] and was modified and used by Yildirim [21] in modeling competency. The instrument involved four sub-constructs consisting of 20 statements with five statements per sub-construct. The sub-constructs of the instrument are awareness (e.g. I am aware of what modeling strategies to use and when to use them to solve an exercise), cognitive strategy (e.g. I attempt to discover the main ideas in an exercise), planning (e.g. I try to understand the goals of an exercise before I attempt to solve it) and self-checking (e.g. I check my accuracy as I progress through the solution). A 5-point scale of strongly disagree (1), disagree (2), uncertain (3), agree (4) and strongly agree (5) was used in the metacognitive inventory questionnaire. Cronbach’s alpha internal consistency reliabilities of the four metacognition sub-constructs (awareness, *α* = 0.83), (cognitive strategy *α* = 0.85), (planning *α* = 0.84) and (self-checking, *α* = 0.83) were above the minimum common cut-off value of *α* > 0.70. All the CR values for the metacognition sub-constructs ranged from 0.83 to 0.85, which exceeded the desirable standard of 0.6. Therefore, a high internal consistency was obtained. Moreover, the AVE for the four latent variables ranged from 0.50 to 0.54, which also exceeded the common cut-off value of 0.5. Therefore, this study achieved acceptable discriminant validity.

## Data analysis

Prior to further analysis, several issues related to data screening, such as handling missing data, multicollinearity and identification of outliers and normality, were considered using Statistical Package for the Social Sciences 23.0 program. Outliers were identified by box plot for each sub-construct. The benchmark of the univariate normality of construct in a measurement model for a latent variable was that the skewness and kurtosis values for each item ranged from −1.96 to 1.96 at 0.05 significant level [112]. Multicollinearity occurs when the correlation matrix with correlations was more than 0.90 [113].

CFA procedures were applied using AMOS 18.0 to investigate whether the established dimensionality and factor-loading pattern fitted Indonesian context. Awang [114] evaluated goodness of fit by using chi-square (χ^2^) (P > 0.05), comparative fit index (CFI > 0.90), Tucker–Lewis index (TLI> 0.90) and root mean square error of approximation (RMSEA < 0.08). Cronbach’s alpha coefficients, composite reliability (CR) and AVE were calculated to determine the reliability of the instrument (total and sub-constructs) and split-half correlations. Alpha values in the current research were not expected to be comparatively high. Hair et al. [112] indicated that exploratory research alpha values of 0.60–0.70 are satisfactory. CR should also be more than 0.60, whereas AVE should be over 0.50 [114].

### Mediator analysis

A mediator (intervening variable) distributes the effect of an independent variable to a dependent variable [115]. The current research used two types of mediator [116]. Firstly, when no significant relationship or direct effect exists between independent variable (achievement goals) and dependent variable (mathematical modeling competency), full mediating effect or complete mediation occurs. Secondly, when a direct effect exists between the causal variable (achievement goals) and the outcome variable (mathematical modeling competency), partial mediating effect or partial mediation happens. To determine the extent of the effect of a mediator on the total effect on the outcome variable, the significance of the indirect effects was determined using AMOS software and Sobel test.

## Results

### Preliminary analysis

The amount of missing data in this research varied from 0% to 0.5% per item. These missing data were completely at random [113]. The means, standard deviations, correlation matrix, skeweness and kurtosis for all variables are shown in Table 1.

**Table 1:** Means, standard deviations, and Pearson correlations between mathematical modeling competency, achievement Goals and sub-construct of metacognition.

Variable	Mathematical modeling competency	Achievement Goals	Awareness	Cognitive Strategy	Planning	Self-Checking
Mathematical modeling competency	1	.456**	.412**	.454**	.421**	.421**
Achievement Goals		1	.427**	.538**	.480**	.481**
Awareness			1	.559**	.486**	.489**
Cognitive Strategy				1	.535**	.475**
Planning					1	.528**
Self-Checking						1
Skew	0.093	-.974	-.133	-.658	-.124	-.154
Kurtosis	-0.136	1.710	.842	2.343	.087	.106
M	0.898	5.296	3.940	3.7372	3.9517	3.910
SD	0.318	.913	.552	.668	.584	.637

Note:

**. Correlation is significant at 0.01 level (2-tailed).

Table 1 shows that the results of preliminary analysis all of the items of the measures of mathematical modeling competency, achievement goals, awareness, cognitive strategy, planning, and self-checking reach univariate normality (skewness and kurtosis values are from -0.974 to 2.343). In terms of multicollinearity, inter-correlations amongst the four metacognition sub-constructs ranged from 0.486 to 0.559, and those for the six achievement goal sub-constructs ranged from 0.473 to 0.611. Therefore, the discriminant validities of the variables were reached because the correlation matrix with correlations was less than 0.90 [114]. The mean value for mathematical modeling competency is (M = 0.898 and SD = 0.318). The mean value for achievement goals is (M = 5.296 and SD = 0.913). The mean values for metacognition vary amongst sub-constructs awareness (M = 3.940 and SD = 0.552), cognitive (M = 3.737 and SD = 0.668), planning (M = 3.951 and SD = 0.584) and self-checking (M = 3.910 and SD = 0.637).

The analysis of the correlations indicated that the correlation between mathematical modeling competency and achievement goals was significant and weak (*r* = .456, *p* < 0.05), mathematical modeling competency and awareness was significant and weak (*r* = .412, *p* < 0.05), mathematical modeling competency and cognitive strategy was significant and weak (*r* = .454, *p* < 0.05), mathematical modeling competency and planning was significant and weak (*r* = .421, *p* < 0.05), mathematical modeling competency and self-checking was significant and weak (*r* = .421, *p* < 0.05), achievement goals and awareness was significant and weak (*r* = .427, *p* < 0.05), achievement goals and cognitive strategy was significant and moderate (*r* = .538, *p* < 0.05), achievement goals and planning was significant and weak (*r* = .480, *p* < 0.05), achievement goals and self-checking was significant and weak (*r* = .481, *p* < 0.05), awareness and cognitive strategy was significant and moderate (*r* = .559, *p* < 0.05), awareness and planning was significant and weak (*r* = .486, *p* < 0.05), awareness and self-checking was significant and weak (*r* = .489, *p* < 0.05), cognitive strategy and planning was significant and moderate (*r* = .535, *p* < 0.05), cognitive strategy and self-checking was significant and weak (*r* = .475, *p* < 0.05), and planning and self-checking was significant and moderate (*r* = .528, *p* < 0.05).

### Testing the measurement models

**Fig 1:**
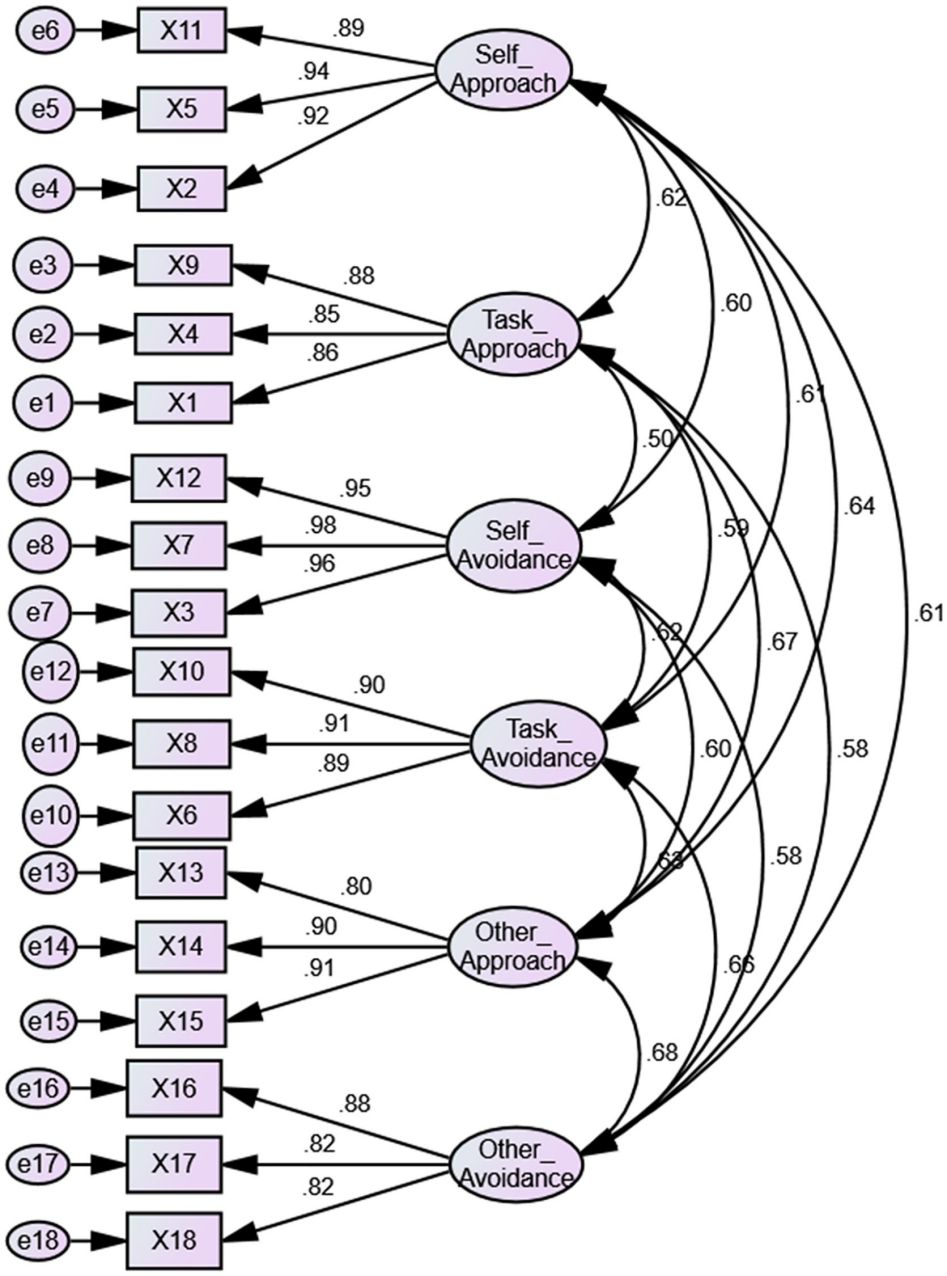
Confirmatory factor analysis path diagram for achievement goals.

CFA procedures were conducted to verify the factorial validity of the four sub-constructs of metacognition and the six sub-constructs of achievement goals. The measurement model of achievement goals resulted in acceptable model fit, χ^2^ = 191.35, χ^2^/*df* = 1.60 CFI = 0.98, TLI = 0.99 and RMSEA = 0.033. Fig 1 was the standardized coefficients acquired from the CFA that indicated the relationships between factors and items for achievement goals.

**Fig 2:**
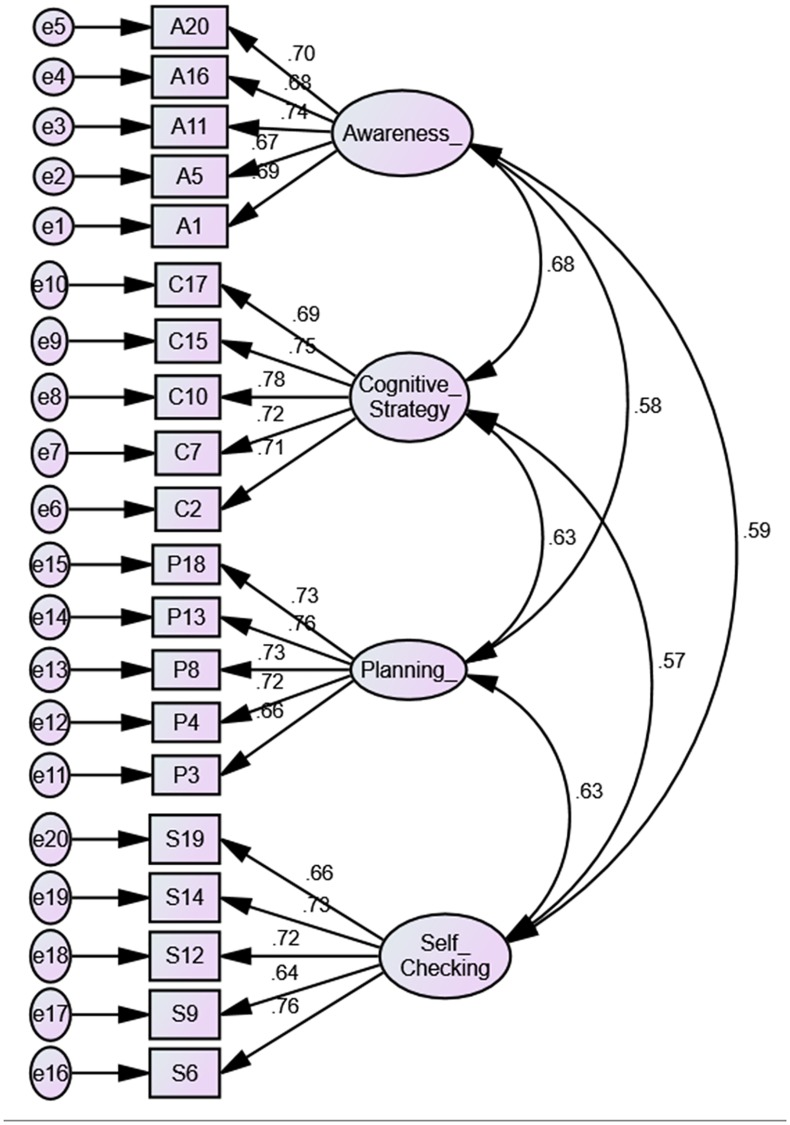
Confirmatory factor analysis path diagram for metacognition.

Fig 1 showed all loadings of items were between 0.80 and 0.98. This exceeded the common cut-off value of 0.5 [112]. In addition, the measurement model of metacognition also indicated acceptable model fit, χ^2^ = 325.454, χ^2^/*df* = 1.98 CFI = 0.97, TLI = 0.96 and RMSEA = 0.043. Fig 2 was the standardized coefficients acquired from the CFA that indicated the relationships between factors and items for metacognition.

**Fig 3:**
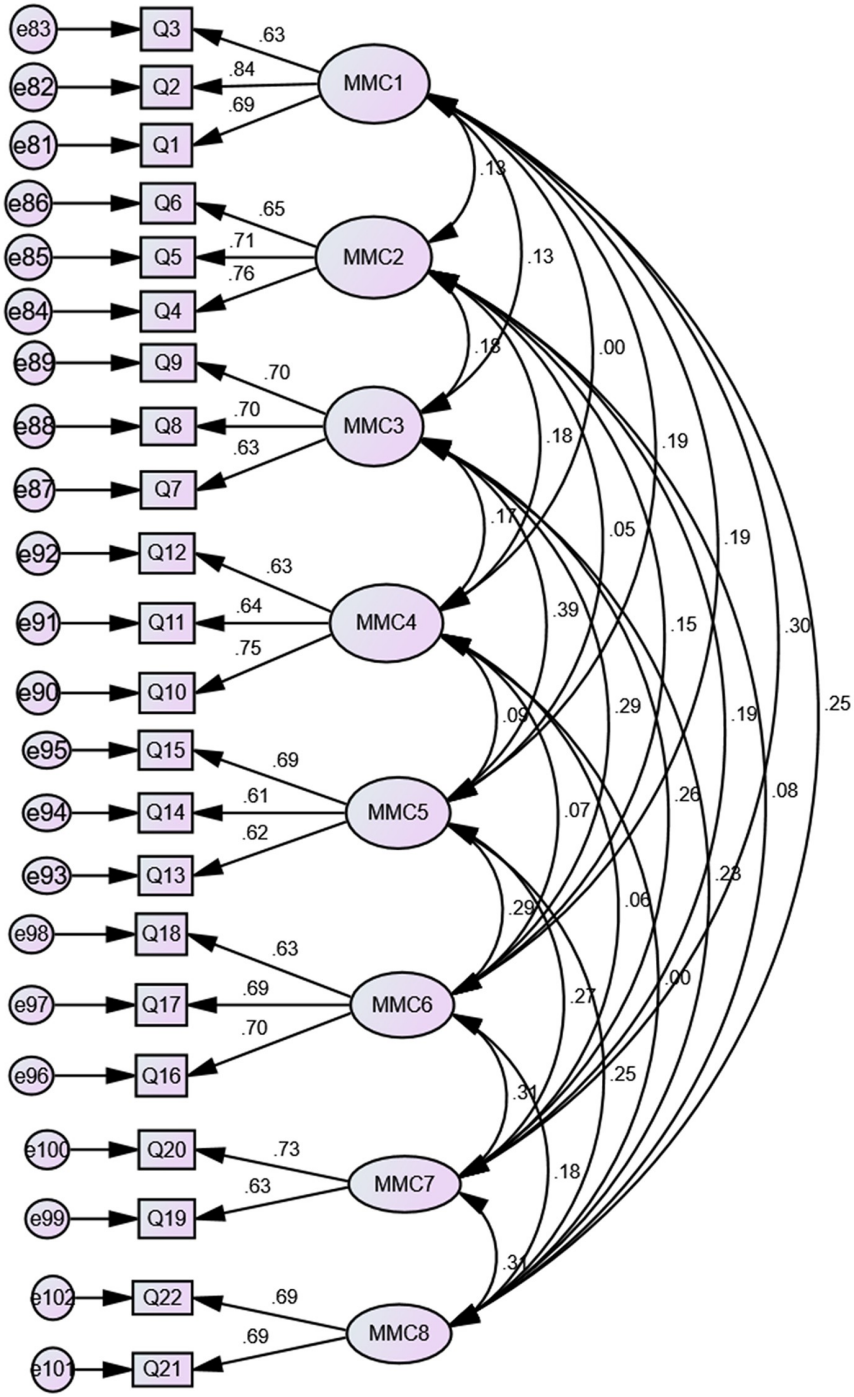
Confirmatory factor analysis path diagram for mathematical modeling.

Fig 2 indicated all loadings of items were between 0.64 and 0.78. This exceeded the common cut-off value of 0.5 [112]. Moreover, the measurement model of mathematical modeling competency also indicated acceptable model fit, χ^2^ = 232.916, χ^2^/*df* = 1.29 CFI = 0.98, TLI = 0.97 and RMSEA = 0.023. Fig 3 was the standardized coefficients obtained from the CFA that indicated the relationships between factors and items for mathematical modeling competency.

Fig 3 indicated all loadings of items were between 0.61 and 0.84. This exceeded the common cut-off value of 0.5 [112].

### Testing the hypothetical structural model

The results of the SEM analysis show the hypothetical structural model of χ^2^ = 2540.561, χ^2^/df = 1.500, RMSEA = 0.031, TLI = 0.948 and CFI = 0.951. All the evaluations resulted in acceptable model fit for the Indonesian context in this research. All factor loadings amongst the four sub-constructs of metacognition ranged from 0.66 to 0.77, and those for the six sub-constructs of achievement goals ranged from 0.80 to 0.98. The factor loading values exceeded the desirable standard of 0.50 [112]. Table 2 shows that the hypothetical structural model is excellent.

**Table 2:** Results of the hypothetical structural model.

Parameter	Coefficient
χ2	2540.561
χ^2^/df	1.500
RMSEA	0.031
TLI	0.948
CFI	0.951

**Note**. χ2: Chi-square goodness of fit; df: Degrees of freedom; CFI: Comparative fit index; TLI: Tucker–Lewis index (TLI); RMSEA: Root mean square error of approximation.

**Fig 4:**
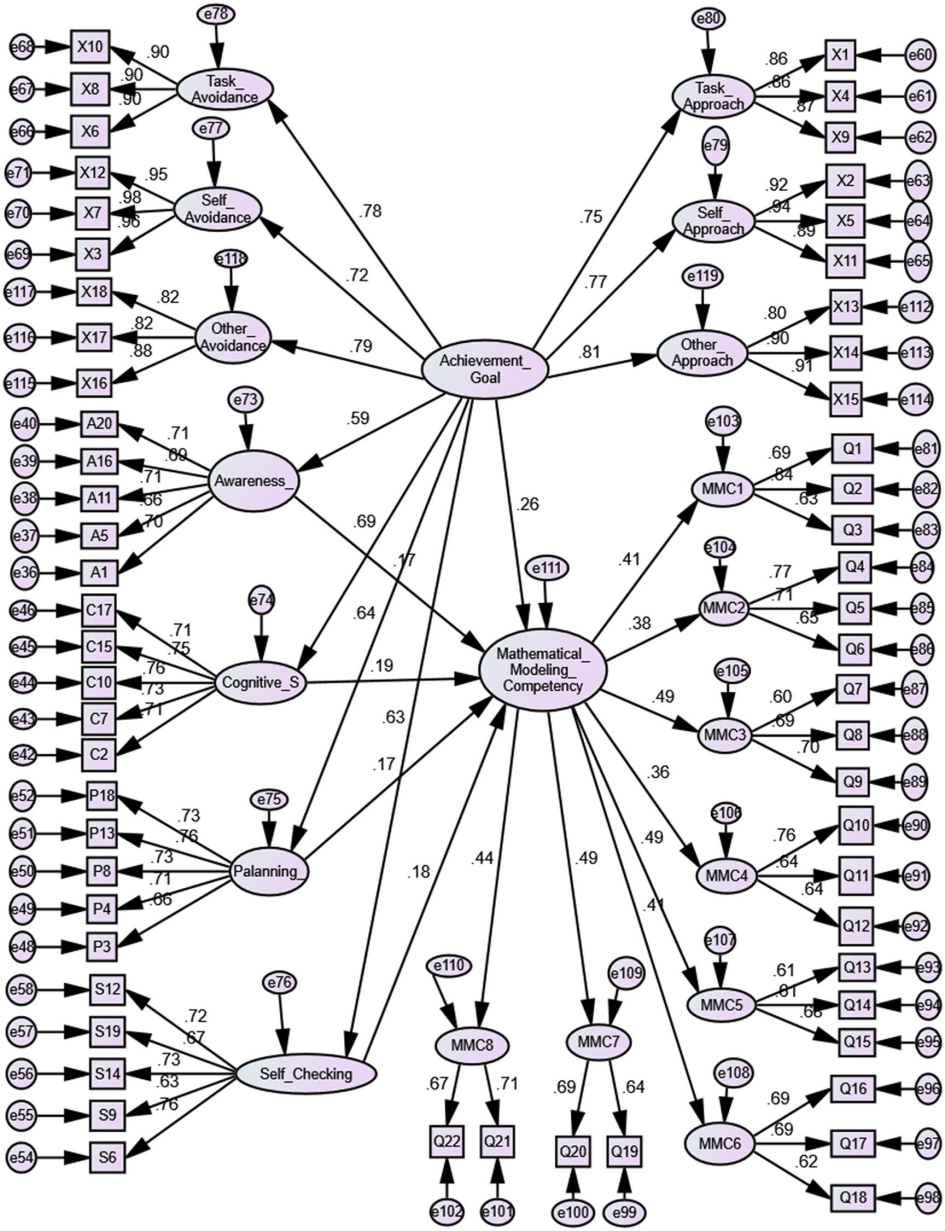
Final model of the study.

Moreover, the model of CFA illustrated in Fig 4 became the finalized model that indicated relationships among achievement goals, metacognition, and mathematical modeling competency in the Indonesian context. The final model derived from the current research can be utilized as an alternative in explaining the previous research on the relationships between achievement goals, metacognition, and mathematical modeling competency.

### Relationships between achievement goals and mathematical modeling competency

We assumed that achievement goals will positively affect mathematical modeling competency. Hypothesis H1 was fully confirmed because significant relationships were found between achievement goals and mathematical modeling competency (β = 0.075, t = 2.09, *p* < 0.05). The findings revealed that students with achievement goals have good performance in mathematical modeling competency. Achievement goals may be a contributing factor towards mathematical modeling competency.

### Relationships among four metacognition sub-constructs (awareness, planning, cognitive strategy and self-checking) and mathematical modeling competency

We hypothesised that four metacognition sub-constructs, namely, awareness, planning, cognitive strategy and self-checking, positively affect mathematical modeling competency. Hypothesis H2 was fully supported because significant relationships were derived between awareness and mathematical modeling competency (β = 0.074, t = 2.30, *p* < 0.05), planning and mathematical modeling competency (β = 0. 078, t = 2.19, *p* < 0.05), cognitive strategy and mathematical modeling competency (β = 0.077, t = 2.33, *p* < 0.05) and self-checking and mathematical modeling competency (β = 0.070, t = 2.39, *p* < 0.05). Students’ awareness, planning, cognitive strategy and self-checking were vital in encouraging students’ mathematical modeling competency.

### Mediating effects of the four metacognition sub-constructs on the relationships between achievement goals and mathematical modeling competency

We expected a mediating effect of awareness, planning, cognitive strategy and self-checking on the relationships between achievement goals and mathematical modeling competency. Table 3 lists the results of mediating effect analysis using AMOS software.

**Table 3:** Output of mediating effect.

Path	Direct effect		Indirect effect		Result
	β	*p* values	β	*p* values	
AG→AW→MMC	0.075	0.037	0.17	0.000	Partial Mediation
AG→CS→MMC	0.075	0.037	0.19	0.000	Partial Mediation
AG→PL→MMC	0.075	0.037	0.17	0.000	Partial Mediation
AG→SC→MMC	0.075	0.037	0.18	0.000	Partial Mediation

Table 3 shows the outputs of the mediating effect of four metacognition sub-constructs, namely, awareness, planning, cognitive strategy and self-checking, on mathematical modeling competency. A positive partial mediating effect of awareness (β = 0.17, *p* < 0.05), cognitive strategy (β = 0.19, *p* < 0.05), planning (β = 0.17, *p* < 0.05) and self-checking (β = 0.18, *p* < 0.05) for achievement goals on mathematical modeling competency (β = 0.26, *p* < 0.05) was found. The mediating effects were tested using the Sobel test analysis to confirm the mediating effect of the four metacognition sub-constructs. Awareness is a significant partial mediator for achievement goals (*z* = 2.04, *p* < 0.05) on mathematical modeling competency. Cognitive strategy is a significant partial mediator for achievement goals (*z* = 2.05, *p* < 0.05) on mathematical modeling competency. Planning is a significant partial mediator for achievement goals (*z* = 2.04, *p* < 0.05) on mathematical modeling competency. Self-checking is a significant partial mediator for achievement goals (*z* = 2.05, *p* < 0.05) on mathematical modeling competency. Overall, achievement goals exert a direct significant effect on mathematical modeling competency (β = 0.26, *p* < 0.05). Thus, hypothesis H3 was fully confirmed.

## Discussion

This study examined the relationship between metacognition and achievement goals which might influence mathematical modeling competency in students of mathematics education programs. Considering the important role of metacognition for mathematical modeling competency in the current literature [29], the mediating effects of four metacognition sub-constructs, namely, awareness, planning, cognitive strategy and self-checking, on achievement goals and mathematical modeling competency have been rarely explored.

### Effects of achievement goals on mathematical modeling competency

The significant direct relationship between achievement goals and mathematical modeling competency was confirmed in our study regardless whether due to task-approach goal, task-avoidance goal, self-approach goal, self-avoidance goal, other-approach goal or other-avoidance goal. Our findings agreed with those of previous research [73, 75]. Students who integrate mastery- and performance-oriented goals support their success in mathematical modeling competency. The reason for this positive relationship is that students who utilised mastery goals were likely to set deep learning [86], effective strategy use [74], perceived competence [73] and material absorption in class [69]. Given that modeling activities also refer to group works to find solutions, mastery goals encourage students’ teacher–student relations, peer inclusion and peer conflict [76]. This deduction was clarified by Liu et al. [71], who found that students who focused on performance goals predict positive learning outcomes, but the relationship was insignificant [94]. The hypothesis relationship between sub-constructs of achievement goals and mathematical modeling competency should be examined in future research because previous findings of relationship amongst sub-constructs of achievement goals and learning results were inconsistent.

### Effects of the four metacognition dimensions (awareness, planning, cognitive strategy and self-checking) on mathematical modeling competency

We found that metacognition positively affected mathematical modeling competency. These results are in line with those of considerable previous research [51, 52, 56]. This relationship may be attributed to metacognition encouraging the use of general strategies such as task analysis, problem representation, prediction, planning, monitoring, checking, reflection and evaluation of success [59] because several mathematical modeling competencies ask students’ ability to simplify assumptions, clarify goals and formulate problems. Moreover, the use of metacognitive strategies allows students to be sensitive in understanding a problem appropriately and make few errors in the learning process, thereby improving their self-regulation skills and self-confidence [60]. Surprisingly, according to the obtained results in the current study, weak relationship exists between mathematical modeling competency and their level of awareness, planning, cognitive strategy and self-checking. Our results partially endorsed prior investigation. No significant correlation between metacognitive self-reported strategy use (planning and monitoring) and mathematical modeling competence [117] were not confirmed in the present research. One possible explanation for the findings may be population we employed. Metacognition increase with age and task specific ability [118] and advanced learners in complicated assignments [119]. Moreover, strategies for regulating and strategies for evaluating among students of grade 9 are low [120]. Thus, the four metacognition dimensions including awareness, planning, cognitive strategy and self-checking in the current research are vital factors in mathematical modeling classroom during modeling cycle.

The current findings showed that the four metacognition dimensions including awareness, planning, cognitive strategy and self-checking partially mediate mathematical modeling competency. These findings are consistent with previous research that found metacognition was a partial mediator [121, 122], and in particular awareness [123], planning [124], cognitive strategy [125] and self-checking [126, 127] as mediators in academic achievement. However, we failed to reiterate a prior study which stated that only three of the four sub-constructs of metacognition (i.e. planning, cognitive strategy and self-checking) significantly affected mathematical modeling competency [21, 128] while awareness was not a mediator toward mathematical modeling competency. Our findings partially endorsed previous research. A possible reason why students’ awareness sometimes works properly towards their mathematical modeling competency in the current research is because metacognition becomes activated in difficult tasks [58]. O’Neil and Abedi [45] emphasized that no metacognition occurs without conscious awareness of it. Therefore, students who utilize great awareness in mathematical modeling classroom are likely to support their planning, cognitive strategy and self-checking. This procedure is vital because students who may fail in mathematical modeling competency if they ignore every single sub-construct of metacognition whilst preparing to participate in a modeling task. In addition, previous studies also have indicated awareness as an insignificant predictor of mathematical modeling competency. Sahin and Kendir [60] found that children usually become successful in problem solving as they are aware of what they need to perform and can supervise procedures.

### Effects of achievement goals on mathematical modeling competency via awareness, planning, cognitive strategy and self-checking

The present study indicates that the four metacognition dimensions including awareness, planning, cognitive strategy and self-checking are positive partial mediators because they improve the association between achievement goals and mathematical modeling competency. The current findings confirm the hypothesis that students with great metacognition are likely to mediate relationship between achievement goals and mathematical modeling competency. These findings support previous research which indicated that metacognition is either a full mediator [93, 94] or a partial mediator [107]. Yildirim [21] found that self-checking, cognitive strategy and planning mediate the development of modeling competencies. Awareness, planning, cognitive strategy and self-checking may be meaningful factors that relate students’ achievement goals and their mathematical modeling competency. The reason for this relationship is that the mediating effects of awareness, planning, cognitive strategy and self-checking effectively control self-regulatory problems during goal striving although students have strong achievement goals [96]. Therefore, students’ awareness, planning, cognitive strategy and self-checking should be supported in mathematical modeling classroom.

## Conclusions and suggestions

The results of this research provide further evidence that achievement goals and metacognition positively influence mathematical modeling competency. In addition, the four metacognition dimensions, namely, awareness, planning, cognitive strategy and self-checking are positive partial mediators. Awareness, planning, cognitive strategy and self-checking in mathematical modeling classroom enhance the relationship between achievement goals and mathematical modeling competency. One practical implication for educators is that they should support students in order to improve their metacognition and achievement goals in solving complex problems in mathematical modeling competencies by providing appropriate learning approaches and offering an adequate learning environment. Moreover, based on the obtained findings, awareness, planning, cognitive strategy and self-checking are positive partial mediators between achievement goals and mathematical modeling competency. This means that besides teachers should encourage students to hold a robust achievement goals, they need to adopt good metacognition in the mathematical modeling class in term of maximizing their achievement in mathematical modeling competency.

Our research has some limitations. First, it is difficult to describe fully of the relationship between two or more variables in correlational study although SEM propose the results about causal relationships. Second, the definition of mathematical modeling competency employed in this study is slightly restricted. The current research involves mathematical modeling competency in a content perspective. Thus, the effect of achievement goals and metacognition towards mathematical modeling competency in modeling as a vehicle should be analyzed. Hence, an experimental study involving mathematical modeling competency as a vehicle should be conducted. Future research also should explore the effect of achievement goal sub-constructs on metacognition and mathematical modeling competency because of inconsistency in previous findings. The role of metacognition sub-constructs in achievement goal sub-constructs and mathematical modeling competency also needs to be examined.

## Supporting information

S1 File Letter of permission from the educational planning and research division. (PDF)

S2 File Data availiability. (XLS)
