# Supplementary material for: Roles of metacognition and achievement goals in mathematical modeling competency: A structural equation modeling analysis
Source: PLoS One. 2018 Nov 6;13(11):e0206211. doi: 10.1371/journal.pone.0206211 (PMC6219774; doi:10.1371/journal.pone.0206211)
Supplement: S1 File — (PDF) [file pone.0206211.s001.pdf]

PEMERINTAH PROVINSI RIAU
DINAS PENANAMAN MODAL DAN PELAYANAN TERPADU SATU PINTU
Gedung Menara Lancang Kuning Lantai I & II Komp. Kantor Gubernur Riau
Jl. Jenderal Sudirman No. 460 Telp. (0761) 39119 Fax. (0761) 39117, PEKANBARU
Email : dpmptsp@riau.go.id Kode Pos : 28126

182010

REKOMENDASI
Nomor : 503/DPMPTSP/NON IZIN-RISET/8323
TENTANG

PELAKSANAAN KEGIATAN RISET/PRA RISET
DAN PENGUMPULAN DATA UNTUK BAHAN DISERTASI

Kepala Dinas Penanaman Modal dan Pelayanan Terpadu Satu Pintu Provinsi Riau, setelah membaca Surat Permohonan Pra Riset dari : **Assistant Registrar (Higher Degrees) Faculty of Education UNIVERSITY MALAYA, Nomor : - Tanggal 29 Agustus 2017**, dengan ini memberikan rekomendasi kepada:

1. Nama	:	**RIYAN HIDAYAT**
2. NIM / KTP	:	PHA 160048
3. Program Studi	:	PENDIDIKAN MATEMATIKA
4. Konsentrasi	:	PENDIDIKAN MATEMATIKA
5. Jenjang	:	S3
6. Judul Penelitian	:	**FAKTOR YANG MEMPENGARUHI KOMPETENSI PEMODELAN MATEMATIK PELAJAR PROGRAM PENDIDIKAN MATEMATIK DI RIAU - INDONESIA**
7. Lokasi Penelitian	:	1. UNIVERSITAS RIAU<br>2. UNIVERSITAS ISLAM RIAU<br>3. UIN SUSKA PEKANBARU<br>4. UNIVERSITAS MUHAMMADIYAH RI<br>5. UNIVERSITAS LANCANG KUNING PEKANBARU<br>6. UNIVERSITAS PASIR PANGARAIAN

Dengan Ketentuan sebagai berikut:

1. Tidak melakukan kegiatan yang menyimpang dari ketentuan yang telah ditetapkan yang tidak ada hubungan dengan kegiatan ini.
2. Pelaksanaan Kegiatan Penelitian dan Pengumpulan Data ini berlangsung selama 6 (enam) bulan terhitung mulai tanggal rekomendasi ini dibuat.

Demikian Rekomendasi ini diberikan agar dapat digunakan sebagaimana mestinya dan kepada pihak yang terkait diharapkan untuk dapat memberikan kemudahan dan membantu kelancaran kegiatan Penelitian dan Pengumpulan Data ini dan terima kasih.

Dibuat di : Pekanbaru
Pada Tanggal : 4 September 2017

a.n. GUBERNUR RIAU
KEPALA DINAS PENANAMAN MODAL DAN PELAYANAN TERPADU SATU PINTU PROVINSI RIAU

EVAREFITA, SE, M.Si
Pembina Utama Muda
NIP. 19720628 199703 2 004

Tembusan :
Disampaikan Kepada Yth :
1. Kepala Badan Kesatuan Bangsa dan Politik Provinsi Riau di Pekanbaru
2. Rektor Universitas Riau di Pekanbaru
3. Rektor Universitas Islam Riau di Pekanbaru
4. Rektor Universitas Islam Negeri Sultan Syarif Kasim Riau di Pekanbaru
5. Rektor Universitas Muhamamdiyah Riau
6. Rektor Universitas Lancang Kuning di Pekanbaru
7. [illegible]
